# Patient Engagement and Symptom Outcomes in a Provider-Guided Online Symptom Management Intervention: Mixed Methods Study

**DOI:** 10.2196/72784

**Published:** 2026-07-23

**Authors:** Yan Wang, Annette DeVito Dabbs, Teresa Hagan Thomas, Grace Campbell, Heidi Ann Donovan

**Affiliations:** 1 Department of Health and Community Systems, School of Nursing, University of Pittsburgh, 3500 Victoria Street, Victoria Building, Pittsburgh, PA, 15261, United States, 1 4126261172; 2 Department of Acute and Tertiary Care, School of Nursing, Univeristy of Pittsburgh, Pittsburgh, PA, United States; 3 School of Nursing, Duquesne University, Pittsburgh, PA, United States; 4 Department of Obstetrics, Gynecology, and Reproductive Sciences, School of Medicine, University of Pittsburgh, Pittsburgh, PA, United States

**Keywords:** engagement, digital health intervention, intervention efficacy, symptom management, eHealth, gynecological cancer

## Abstract

**Background:**

Cancer survivors often experience declining engagement in digital health interventions (DHIs). However, predictors of engagement with provider-guided DHIs remain unclear. Nurse WRITE (Nurse Written Representational Intervention To Ease Symptoms), an effective 8-week nurse-directed symptom management DHI, offers an opportunity to identify factors influencing engagement and enhance intervention efficacy evaluation.

**Objective:**

This study aims to (1) understand engagement phenomena (dimensions, influencing factors, and challenges), and (2) assess the relationship between engagement and patient symptom control in Nurse WRITE.

**Methods:**

In this secondary analysis of the Nurse WRITE arm of a 3-arm symptom management trial, we examined data from 68 women with recurrent ovarian cancer to assess socioaffective and cognitive engagement through message board activity, as well as behavioral engagement through website usage data. Regression analyses examined patient characteristics, engagement, and symptom control perceptions. Through content analysis, we explored participant challenges and activities before disengagement.

**Results:**

Education was significantly associated with selected cognitive, socioaffective, and behavioral engagement indicators, including cognitive activity count, total word count, completion of symptom care plans, and plan reviews after false discovery rate correction. The most common engagement challenges included worsening health and treatment, busy family life, and website difficulties. Moderate and low engagers also experienced confusion about the intervention timeline and process. Among low engagers, 63.2% (24/38) discontinued communication at specific intervention phases: introduction (8/24, 33.3%), symptom representational assessment (5/24, 20.8%), and goal setting and planning (5/24, 20.8%). Improved symptom control at the end of the intervention was significantly associated with overall engagement, cognitive and socioaffective activity count, question completion percentage, total word count, and completed symptom care plans after false discovery rate correction.

**Conclusions:**

Education was associated with selected cognitive, socioaffective, and behavioral engagement indicators in Nurse WRITE. Future provider-guided DHIs should consider strategies to support participants with lower educational attainment, address common engagement barriers, and reengage participants during critical intervention phases. Meaningful engagement across cognitive, socioaffective, and behavioral dimensions may be important for improving outcomes while balancing protocol adherence with flexibility.

## Introduction

In 2024, an estimated 19,680 women will be diagnosed with ovarian cancer, and approximately 12,740 will die [1]. Despite aggressive surgery and an initial chemotherapy response, 85% of these women will experience recurrence within 2 years [2,3]. The focus then shifts to balancing the potential of life-extending treatments with multiple severe symptoms, requiring novel interventions to improve patients’ symptoms and quality of life.

In the past decade, digital technologies like mobile health and eHealth have been increasingly integrated into interventions for cancer survivors, addressing symptom management [4,5] and promoting healthy behaviors [6-8]. Active engagement with these technologies is crucial for effective symptom management and improved outcomes [9]. However, declining engagement with digital health interventions (DHIs) over time has been widely observed [10,11].

Previous studies have examined patient characteristics (eg, age, employment, education, and marital status [9,12,13]), social support [14,15], self-efficacy [16], and clinical factors (eg, comorbidities [13,17], well-being [18], distress, depression, and anxiety [17-20]) associated with engagement in DHIs. However, research on how these factors influence engagement in provider-guided DHIs, which include ongoing human support and interpersonal interaction, remains limited. Unlike self-guided DHIs, provider-guided interventions involve individualized feedback and therapeutic relationships that may shape engagement patterns differently [21-23]. Disengagement in these interventions may reflect not only technology usability issues but also emotional distress, symptom burden, or evolving care needs. Participants may also disengage temporarily and later reengage, making engagement a dynamic and complex process that is difficult to capture using traditional usage metrics. Understanding engagement patterns in provider-guided DHIs is therefore critical to optimizing intervention delivery and improving outcomes.

Nurse WRITE (Nurse Written Representational Intervention To Ease Symptoms), a nurse-guided DHI, was one intervention arm of a 3-arm online randomized clinical trial designed to help women with recurrent ovarian cancer manage symptoms [4]. Using the Representational Approach to patient education [24], nurse interventionists assessed participants’ symptom representations (beliefs and understandings about cause, timeline, consequences, controllability, and emotional impact) and developed individualized care plans through asynchronous message boards. The nurses also guided participants in developing problem-solving skills for managing symptoms following the 8-week intervention. The comparison arm (SD WRITE) consisted of self-directed online modules without nurse interaction. A key outcome was perceived control over symptoms, reflecting confidence, persistence in problem-solving, and behavioral orientation [25], which predicts long-term outcomes [26-28]. The Nurse WRITE intervention significantly improved symptom control compared to the enhanced usual care arm [4].

Given the importance of engagement in provider-guided DHIs, evaluating patient engagement in Nurse WRITE and its relationship to perceived symptom control is critical for understanding intervention effectiveness and informing translation into clinical practice.

Previous Nurse WRITE papers have made 2 important contributions. First, the parent trial evaluated the efficacy of Nurse WRITE and demonstrated that the nurse-guided intervention improved perceived symptom control compared with enhanced usual care [4]. Second, subsequent methodological work conceptualized engagement in provider-guided DHIs and developed an analytic framework and tool for assessing socioaffective, cognitive, and behavioral engagement within asynchronous nurse-patient communication [29,30]. However, these prior studies did not empirically examine which patient and clinical characteristics are associated with different engagement dimensions, what challenges impede engagement, or whether engagement dimensions are associated with intervention outcomes.

This study addresses this gap by applying the previously developed engagement framework and analytic tool to Nurse WRITE trial data to empirically evaluate engagement processes in a provider-guided DHI. Specifically, this study aims to (1) identify baseline patient characteristics, including age, education, employment, marital status, optimism, and social support, and clinical characteristics, including anxiety, depressive symptoms, comorbidities, symptom severity, controllability, consequences, and distress, that are associated with engagement levels and challenges impeding engagement and (2) examine the relationship between socioaffective, cognitive, and behavioral engagement and perceived symptom control at the end of the intervention. Findings will clarify how engagement operates in provider-guided DHIs and inform strategies for tailoring intervention delivery to enhance learning, behavior change, and symptom management outcomes.

## Methods

### Design

This secondary observational analysis had 2 components. First, using a convergent mixed methods approach [31], we explored patient and clinical characteristics influencing engagement through quantitative analysis and identified challenges and predisengagement activities on asynchronous message boards via qualitative analysis. Quantitative and qualitative findings were integrated using side-by-side comparison and triangulation, whereby qualitative themes were used to explain and contextualize quantitative associations related to engagement. This integration provided both statistical insights and rich contextual nuances, offering a holistic view of engagement phenomena. Second, we conducted statistical analysis to assess the relationship between engagement and patient-perceived symptom control at the intervention’s end. This ancillary analysis is reported in accordance with the STROBE (Strengthening the Reporting of Observational Studies in Epidemiology) guidelines [32], and the mixed methods components in accordance with the GRAMMS (Good Reporting of a Mixed Methods Study) guidelines [33].

### Ethical Considerations

The WRITE symptom interventions were approved by the University of Pittsburgh Institutional Review Board (PRO09090033) and 34 clinical sites, allowing deidentified data sharing [4]. All activities and questionnaires were conducted on a secure, HIPAA (Health Insurance Portability and Accountability Act)-compliant website developed at the University of Pittsburgh. This secondary data analysis used deidentified data, posing no risk to participants.

### Sample and Nurse WRITE Asynchronous Message Boards

The parent trial was conducted from January 2010 to January 2017 (ClinicalTrials.gov NCT00958698) across NRG Oncology/Gynecologic Oncology Group–affiliated sites [4]. Of the 497 women with recurrent ovarian cancer enrolled in the parent trial, 166 were assigned to the Nurse WRITE arm. Among these participants, 141 posted at least one message on the intervention message board and were therefore eligible for engagement analysis. This study included the first 68 eligible participants, representing 48.2% (68/141) of the eligible message-board participants and approximately 50% of the message-board content. This sample was selected because the engagement analysis required intensive manual coding of asynchronous nurse-participant communication using the multidimensional engagement framework, including socioaffective, cognitive, and behavioral engagement and engagement challenges. This process involved a detailed review and classification of each participant’s message-board interactions; therefore, coding the full eligible Nurse WRITE sample was not feasible within the scope of this study.

To assess potential selection bias, we compared baseline patient and clinical characteristics between the analytic sample and the remaining eligible Nurse WRITE participants who posted at least one message. No statistically significant group differences were observed in demographic, psychosocial, or symptom-related variables, suggesting that the analytic sample was broadly comparable to the larger eligible Nurse WRITE sample.

In Nurse WRITE, participants and nurses used private message boards to cocreate personalized symptom care plans for 3 target symptoms. These interactions included open-ended prompts and protocolized questions. The message boards also captured unprompted patient narratives, including challenges faced during the intervention and activities performed before disengagement.

### Measures

#### Participant Data

Patient characteristics included sociodemographic characteristics, optimism, and social support. Clinical characteristics measured at baseline included comorbidities, depressive symptoms, trait anxiety, symptom severity, controllability, consequences, and distress. Sociodemographic factors (age, education, marital status, and employment) were assessed with the Center for Research in Chronic Disorders Sociodemographic Survey [34]. Optimism was measured by the reliable and validated Revised Life Orientation Test [35], an 8-item scale. Items were rated from 0 (agree a lot) to 4 (disagree a lot). The scores were aggregated to a total score. Social support was measured by the validated Interpersonal Support Evaluation List 12-item short form [36]. Items were rated from 1 (definitely false) to 4 (definitely true) and summed up for a total score. Comorbidities were assessed using the validated Charlson Comorbidity Index, a 10-item self-report comorbidity index with each comorbidity weighted based on 1-year mortality risk [37]. Trait anxiety was assessed with the validated 20-item trait anxiety subscale of the Spielberger State-Trait Anxiety Inventory [38], which evaluates how a participant feels on a 4-point scale (1=“Almost Never” to 4=“Almost always”) and is summed for an overall score. Depressive symptoms were assessed with the Brief Center for Epidemiologic Studies Depression scale [39]. Ten items were rated on a 4-point scale (0=“rarely or none of the time” to 3=“All of the time”) and summed for a total score. Symptom severity, consequences, distress, and controllability were assessed with the Symptom Representation Questionnaire, which is a validated scale of symptom representations in women with ovarian cancer [40]. First, participants completed a 28-item symptom inventory reporting symptom severity (at its worst) in the past week from 0 (did not experience the symptom) to 10 (as bad as I can imagine). Second, participants identified 3 symptoms they wanted to improve or control. Another 3 subscales assessed consequences (5 items), distress (3 items), and controllability (5 items) for each target symptom on a scale of 0 (strongly disagree) to 4 (strongly agree). The mean scores for symptom representation items across 3 “target” symptoms at baseline were used for the statistical analysis in aim 1. Additionally, for aim 2, we also assessed the mean score for symptom controllability, our primary intervention outcome variable, at the 8-week mark. This measurement involved averaging the total scores of 5 items related to each of the 3 “target” symptoms. These items included statements like “I have a significant degree of control over this symptom” and “My actions can influence whether this symptom improves or worsens.”

#### Engagement Measures and Engagement Patterns

Table 1 presents the engagement analysis framework, which includes 6 measures across socioaffective, cognitive, and behavioral dimensions, as developed in our prior work [30]. Consistent with this framework, engagement was conceptualized as a multidimensional construct rather than as equivalent to intervention dose or completion. Behavioral indicators, such as question completion and care plan completion, were included as one established dimension of engagement; however, the engagement classification also incorporated socioaffective and cognitive indicators derived from message-board communication. Thus, engagement in this study reflected not only the amount of intervention activity completed but also the nature and quality of participants’ interaction with the intervention content and the nurse interventionist.

Participants were classified into high, moderate, and low engagement groups using k-means clustering based on the 6 engagement measures shown in Table 1. The clustering analysis identified 3 distinct engagement groups: high engagers (n=13), moderate engagers (n=17), and low engagers (n=38). Because the engagement measures were nonnormally distributed, medians and IQRs were used to describe engagement levels within each group. Additional details on the development of the engagement framework, measures, and clustering approach are provided in our prior work [30].

**Table 1.** Description of 6 engagement measures across 3 dimensions.

Engagement dimension and engagement measure	Description
Socioaffective	
Total count of socioaffective classes	A summary score of 7 socioaffective engagement classes (ie, intervention information, acknowledgment, information-seeking question, clarification question, agreement, disagreement, and initiative-taking).
Total word count	Total word count in participants’ posts throughout the intervention.
Cognitive	
Total count of cognitive classes	A summary score of 8 cognitive engagement classes (ie, positive sentiment, negative sentiment, appreciation, cancer-related experience, personal information, vocatives, interest in further communication, and greetings).
Average question completion percentage	The percentage of the nurse’s questions and requests that each participant addressed.
Behavioral	
Total count of symptom care plans	Total count of symptom care plans participants cocreated with the nurse.
Total count of plan reviews	Total count of plans reviewed and revised by participants.

### Analysis

#### Aim 1: Understanding Engagement Phenomena (Dimensions, Influencing Factors, and Challenges)

Preliminary bivariate analyses were conducted to identify candidate baseline patient and clinical variables associated with engagement outcomes. A screening threshold of *P*≤.20 was used to identify potential variables for multivariable model building. These bivariate analyses were used only for variable screening and were not interpreted as primary inferential findings.

Candidate variables were entered into multivariable models, and final models were selected using the minimum Akaike Information Criterion. Ordinal regression was used to examine baseline patient and clinical characteristics associated with overall engagement level, categorized as high, moderate, or low. Multiple linear regression was then used to examine baseline patient and clinical characteristics associated with engagement across the socioaffective, cognitive, and behavioral dimensions, as represented by the 6 engagement measures. Statistical significance was initially evaluated at *P*<.05.

To address multiple testing, the Benjamini-Hochberg false discovery rate (FDR) correction was applied within each aim to the *P* values from the final models. For aim 1, the correction was applied to the *P* values for predictors retained in the final ordinal regression model for overall engagement level and the final linear regression models for the 6 engagement measures. For aim 2, FDR correction was applied to the *P* values for engagement predictors examined in relation to perceived symptom controllability at 8 weeks. For both aims, unadjusted and FDR-adjusted *P* values were examined, and findings were interpreted in light of the adjusted results.

We conducted a content analysis of 68 participants’ posts on the message boards to summarize the challenges to engagement mentioned by participants without nurse prompts during the intervention. Additionally, for the 39 participants who discontinued participation on the message boards during the intervention, 2 investigators (YW and R DiLello) examined the specific intervention elements participants were working on before they stopped responding. Interrater reliability was assessed based on the posts from these 39 participants, with a Cohen κ of 0.98. The intervention elements and goals are available in Multimedia Appendix 1.

#### Aim 2: Relationship Between Patient Engagement Levels and Symptom Controllability

After listwise deletion, 66 participants were included in the analysis for aim 2. Two participants were excluded because their symptom controllability score at the end of the program was missing; no other analysis variables had missing data. Multiple linear regression was used to examine the relationship between perceived symptom controllability at 8 weeks and engagement across the 3 dimensions, represented by the 6 engagement measures, after controlling for baseline symptom controllability and other retained patient and clinical covariates. Patient and clinical covariates were determined based on preliminary bivariate analyses with perceived symptom controllability at 8 weeks, using a screening threshold of *P*≤.20. Statistical significance was initially evaluated at *P*<.05.

For aim 2, the Benjamini-Hochberg FDR correction was applied to *P* values for engagement predictors examined in relation to perceived symptom controllability at 8 weeks. For both aims, unadjusted and FDR-adjusted *P* values were examined, and findings were interpreted in light of the adjusted results.

## Results

### Sample Characteristics

**Figure 1.**
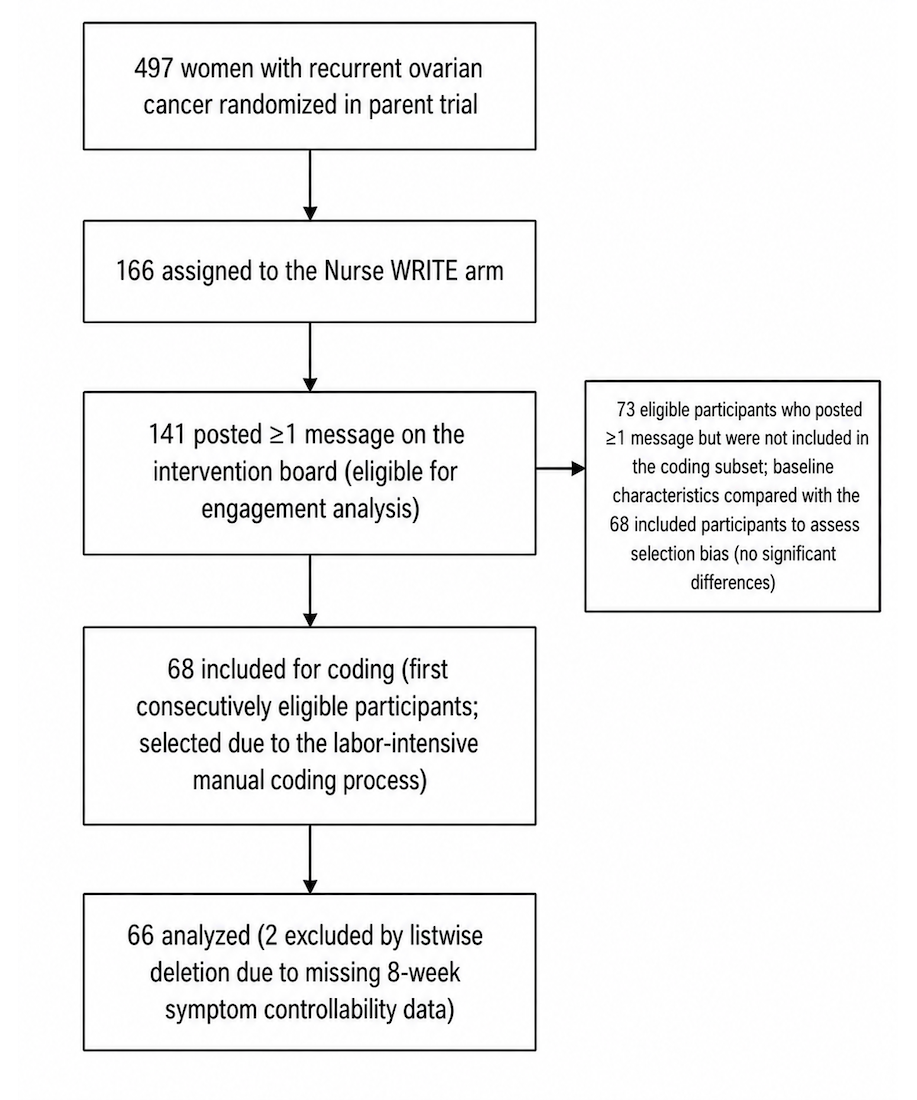
Participant flow for the ancillary analysis of nurse WRITE (Written Representational Intervention To Ease) participants.

Table 2 provides data on baseline patient and clinical characteristics. On average, participants were 59.7 (SD 9.5) years old and had 15.3 (SD 2.6) years of formal education. Most were White (63/68, 92.6%) and married or cohabitating (51/68, 75%). Over half of the participants (37/68, 54.4%) were unemployed due to disability, retirement, or unemployment. Across 3 target symptoms, symptom-related factors revealed moderate mean severity (5.3), controllability (2.2), distress (2.2), and low consequences (2.0). Over half (38/68, 55.9%) had at least 1 comorbidity (eg, hypertension and diabetes). Figure 1 presents the participant flow for this ancillary analysis.

**Table 2.** Patient and clinical characteristics at baseline (N=68).

Characteristics	Value
Age (y), mean (SD; range)	59.7 (9.5; 24‐83)
Education, mean (SD; range)	15.3 (2.6; 11‐22)
Employment, n (%)	
Working	24 (35.3)
Not working	37 (54.4)
Other	7 (10.3)
Marital status, n (%)	
Married/cohabitant	51 (75)
Other	17 (25)
Trait anxiety, mean (SD; range)	36.7 (9.2; 20‐80)
Depression, mean (SD; range)	8.3 (5.7; 0‐30)
Social support, mean (SD; range)	40.6 (6.7; 12‐48)
Optimism, mean (SD; range)	22.8 (4.2; 0‐32)
Symptom severity, mean (SD; range)	5.3 (2.2; 0‐10)
Symptom controllability, mean (SD; range)	2.2 (0.7; 0‐4)
Symptom consequences, mean (SD; range)	2.0 (0.6; 0‐4)
Symptom distress, mean (SD; range)	2.2 (0.7; 0‐4)
Comorbidity, n (%)	
Yes	38 (55.9)
No	30 (44.1)

### Aim 1: Understanding Engagement Phenomena (Dimensions, Influencing Factors, and Challenges)

#### Relationship Between Patient and Clinical Factors and Engagement Levels in 3 Dimensions

Tables 3 and 4 present the ordinal and multiple linear regression models examining patient and clinical characteristics associated with engagement. After FDR correction, education was no longer significantly associated with overall engagement level (odds ratio [OR] 1.22, unadjusted *P*=.04; FDR-adjusted *P*=.08).

In the socioaffective dimension, higher education was associated with a greater word count, and this association remained at the FDR-adjusted significance threshold (B=1467.8, unadjusted *P*=.02; FDR-adjusted *P*=.05). Additionally, higher education (B=2.44, unadjusted *P*=.07; FDR-adjusted *P*=.09) and the absence of comorbidities (B=13.32, unadjusted *P*=.06; FDR-adjusted *P*=.09) showed exploratory trends toward more frequent socioaffective engagement activities.

In the cognitive dimension, education was positively associated with cognitive engagement and remained at the FDR-adjusted significance threshold (B=1.89, unadjusted *P*=.01; FDR-adjusted *P*=.05). The absence of comorbidity showed an exploratory trend but was not statistically significant after correction (B=7.13, unadjusted *P*=.07; FDR-adjusted *P*=.09). No patient or clinical factors were associated with the average question completion percentage.

In the behavioral dimension, education remained associated with completing more symptom care plans (OR 1.23, unadjusted *P*=.02; FDR-adjusted *P*=.05) and care plan reviews (OR 1.29, unadjusted *P*=.01; FDR-adjusted *P*=.05).

**Table 3.** Logistic regression models of patient and clinical characteristics predicting engagement in women with recurrent ovarian cancer (N=66).

Engagement outcome and predictor	B^a^ (SE)	OR^b^ (95% CI)	*P* value^c^	FDR^d^-adjusted *P* value^e^	McFadden *R*^2f^	Model *P* value^g^
High vs moderate vs low engagers						
Education	0.20 (0.10)	1.22 (1.01-1.50)	.04	.08	0.05	.006
Comorbidity (yes vs no)	−0.78 (0.50)	0.46 (0.17-1.20)	.12	.12	0.05	.006
Total count of symptom care plans						
Education	0.21 (0.09)	1.23 (1.04-1.47)	.02	.05	0.03	.008
Total count of plan reviews						
Education	0.26 (0.10)	1.29 (1.07-1.59)	.01	.05	0.07	.008
Symptom severity at baseline	0.19 (0.11)	1.21 (0.97-1.52)	.09	.10	0.07	.008

a B: unstandardized coefficient.

b OR: odds ratio.

c *P* value for the model.

d FDR: false discovery rate.

e *P*: FDR-adjusted *P* value.

f McFadden *R*2: McFadden pseudo *R*-squared for the model.

g *P* value for the independent variable.

**Table 4.** Linear regression models of patient and clinical characteristics predicting engagement in women with recurrent ovarian cancer (N=66).

Engagement outcome and predictor	B^a^ (SE)	β^b^ (95% CI)	*P* value^c^	FDR^d^-adjusted *P* value^e^	Adjusted *R*^2^^f^	Model *P* value^g^
Total count of socioaffective classes						
Education	2.44 (1.33)	0.22 (−0.21 to 5.08)	.07	.09	0.06	.04
Comorbidity (≥1)	−13.32 (6.96)	−0.23 (−27.23 to 0.59)	.06	.09	0.06	.04
Total word count						
Education	1467.8 (647.2)	0.27 (174.99 to 2760.65)	.02	.05	0.06	.03
Total count of cognitive classes						
Education	1.89 (0.74)	0.30 (0.41 to 3.37)	.01	.05	0.10	.02
Comorbidity (≥1)	−7.13 (3.89)	−0.22 (−14.89 to 0.64)	.07	.09	0.10	.02

a B: unstandardized coefficient.

b β: standardized coefficient.

c *P* value for the independent variable.

d FDR: false discovery rate.

e *P*: FDR-adjusted *P* value.

f Adj *R*2: adjusted *R*-squared for the model.

g *P* value for the model.

#### Engagement Challenges and Participants’ Activities Before Disengagement

**Figure 2.**
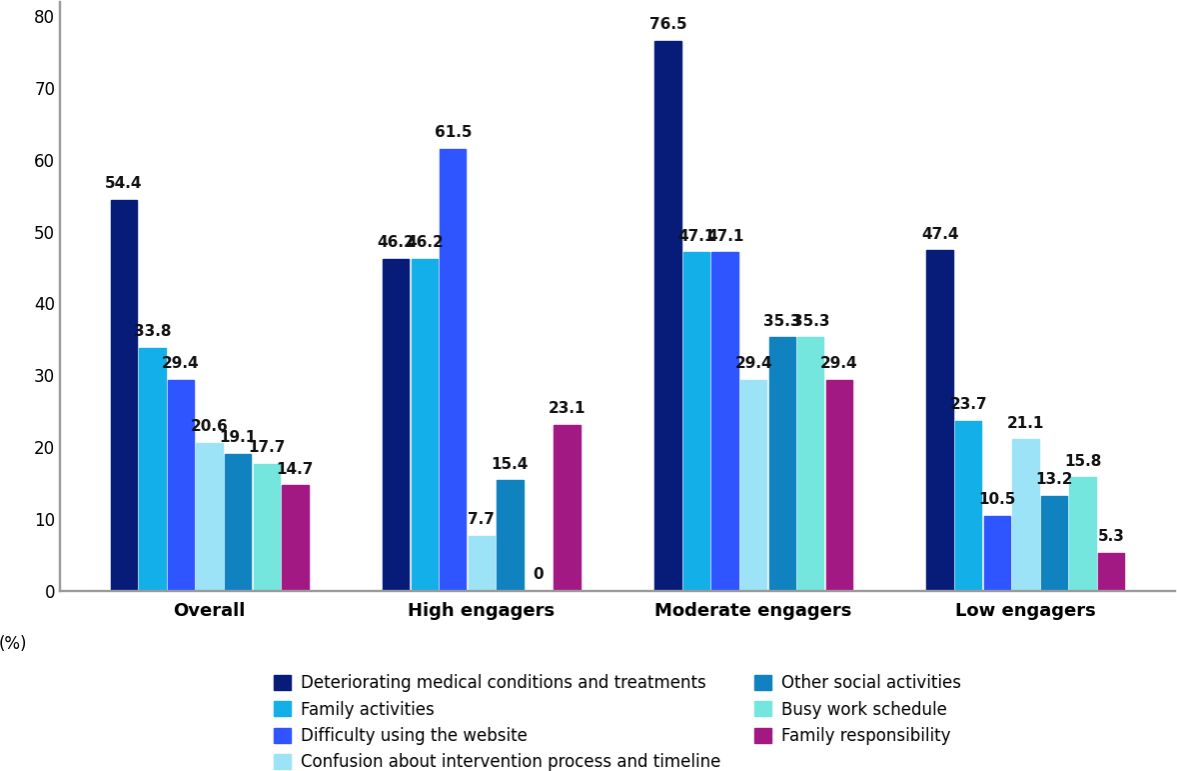
Most frequent challenges faced by engagers (N=68).

Across all engagement levels, the most common challenges to engaging in the intervention, reported by 54.4% (37/68) of participants, were related to deteriorating medical conditions and treatments. These included new or worsening symptoms, infections, surgeries, hospitalizations, cancer treatments, medical appointments, and clinical trials. The second significant challenge, cited by 33.8% (23/68) of participants, was being occupied with family activities such as vacations, visits, celebrations, reunions, funerals, and sports. The third challenge, noted by 29.4% (20/68) of participants, involved difficulties using the website or message boards, such as locating documents or reading nurses’ posts. Figure 2 depicts the most frequently endorsed challenges for engagers as a whole and within each group. Other less prevalent challenges reported by engagers encompassed limited computer literacy and internet and hardware issues.

Considering engagement challenges by group, high engagers faced the same top 3 challenges, albeit in a different order. Besides the same common challenges, busy work schedules (6/17, 35.3%) and other social activities (6/17, 35.3%) were frequently cited for moderate engagers. Compared to high engagers, a significant portion of moderate engagers (5/17, 29.4%) felt confused about the intervention process and timeline and mentioned challenges related to family responsibilities.

Low engagers who mentioned challenges, apart from declining health (18/38, 47.4%) and family activities (9/38, 23.7%), also reported confusion about the intervention (8/38, 21.1%), similar to some moderate engagers. Table 5 presents the intervention activities completed by all 38 low engagers. Among these 38 low engagers, 24 (63.2%) stopped participating on the message boards before the intervention ended. In contrast, none of the high engagers stopped responding, and only 1 moderate engager stopped responding when asked by the nurse to select symptom management strategies.

**Table 5.** The percentage of pre-disengagement intervention activities of low engagers in the order of nurse WRITE intervention elements (N=38).

Intervention phase	Participants, n (%)
Introduction	8 (33.3)
Representational assessment	5 (20.8)
Addressing concerns with new information	3 (12.5)
Choosing strategies and setting goals	5 (20.8)
Reviewing goals and strategies	3 (12.5)

### Aim 2: Relationship Between Patient Engagement Levels and Symptom Controllability

The engagement pattern regression model showed significant results (*F*_7,48_=7.05, adj *R*^2^=0.44, *P*=.008). Higher overall engagement (high vs moderate vs low) was associated with improved symptom control at the end of the intervention (B=0.44, *P*=.02; FDR-adjusted *P*=.03), after controlling for baseline symptom controllability and sociodemographic factors.

In the socioaffective dimension, both the model for the total count of socioaffective classes (*F*_6,49_=7.94, adj *R*²=0.43, *P*=.008) and the model for total word count (*F*_6,49_=8.12, adj *R*²=0.44, *P*=.009) yielded significant results. Improved symptom control at the end of the intervention was associated with greater engagement in activities (B=0.27, *P*=.01, FDR-adjusted *P*=.03) and higher word counts in participants’ posts (B=0.28, *P*=.01, FDR-adjusted *P*=.03), controlling for covariates.

In the cognitive dimension, both the model for the total count of cognitive classes (*F*_6,49_=7.7, adj *R*²=0.42, *P*=.009) and the model for average question completion percentage (*F*_6,49_=9.61, adj *R*²=0.48, *P*=.01) yielded significant results. After adjusting for covariates, improved symptom control at the end of the intervention was associated with greater engagement in activities (B=0.28, *P*=.02, FDR-adjusted *P*=.03) and a higher percentage of questions addressed by participants (B=0.35, *P*=.007, FDR-adjusted *P*=.008).

In the behavioral dimension, only the model for symptom care plan count was significant (*F*_6,49_=6.47, adj *R*²=0.37, *P*=.009). After adjusting for covariates, completing more symptom care plans was significantly associated with better symptom control at the end of the intervention (B=0.25, *P*=.05).

## Discussion

### Principal Results

#### Principal Findings

This study examined patient and clinical characteristics associated with engagement in Nurse WRITE, engagement challenges, and the relationship between engagement and perceived symptom controllability. Three main findings emerged. First, education was the most consistent patient characteristic associated with specific engagement measures. Second, qualitative findings contextualized engagement challenges, including intervention complexity, written communication demands, symptom burden, competing responsibilities, and early disengagement. Third, higher overall engagement and greater socioaffective and cognitive engagement were associated with improved symptom controllability at the end of the intervention.

#### Aim 1: Understanding Engagement Phenomena (Dimensions, Influencing Factors, and Challenges)

Quantitative findings identified education level as the most consistent predictor of specific engagement measures, consistent with a prior review [12]. Qualitative analysis further explained this association, suggesting that participants with lower education levels may have struggled with the length and complexity of nurses’ messages, which is the primary mode in Nurse WRITE. For example, 20% to 30% of less engaged participants, compared to high engagers, reported confusion about the intervention process, even though nurses had explained it at the outset. These challenges may also reflect differences in health literacy, communication preferences, or other linguistic factors, which could influence participants’ ability to engage with written intervention materials [41]. Future research should further explore how these factors intersect with education level to affect engagement in DHIs.

Compared to SD WRITE, where participants interacted with self-directed modules without nurse involvement, the presence of nurses in Nurse WRITE may have influenced how less well-educated patients engaged with the intervention. Participants might have felt self-conscious about their writing when communicating with nurses and faced challenges such as website navigation and confusion regarding the intervention process. Previous studies have linked computer literacy and DHI engagement positively [42-44], but none, including ours, adequately measured baseline computer literacy. Therefore, future provider-guided DHIs should incorporate this measure into their designs to confirm its impact.

Although nurses were encouraged to tailor their responses to participants’ capacities and needs, they faced challenges adhering to intervention protocols. For instance, while some participants preferred phone communication, nurses were restricted to asynchronous message boards. Additionally, nurses were required to follow structured intervention phases, even when some participants wanted to skip directly to solutions, bypassing representational assessments or discussions of concerns (eg, opioid addiction). While the relationship between lower education and lower engagement warrants further investigation, identifying education level as a potential risk factor for reduced engagement with specific intervention activities could help target participants who may need additional support.

Quantitative findings showed an exploratory pattern, suggesting that patients with higher symptom severity may have completed more care plan reviews, which could reflect motivation to revisit and refine symptom management strategies. However, qualitative findings indicated that deteriorating health and ongoing treatments posed significant challenges, often requiring hospitalization or medical appointments that limited participants’ ability to remain engaged. Together, these findings suggest that symptom burden may both motivate and hinder engagement.

Additionally, quantitative findings showed exploratory trends, suggesting that comorbidities, such as hypertension and diabetes, may pose additional challenges for patients with recurrent ovarian cancer and may affect participation. These findings align with previous research on engagement in DHIs [12]. Complementing these results, qualitative data revealed that participants often prioritized managing their health over engaging in symptom management activities on the message boards. Beyond health-related challenges, competing responsibilities, including demanding work schedules, family obligations, and other social commitments, also reduced participants’ time and energy for learning and managing symptoms. Future research with larger sample sizes is needed to confirm the impact of these factors.

Lastly, qualitative findings indicated that over half of the low engagers stopped participating after the introduction and representational assessment phase. This early disengagement may reflect challenges in establishing trust and rapport, underscoring the importance of relationship building during the orientation phase [45]. Early video conferencing for introductions and orientation allows nurses to gather verbal and nonverbal cues related to symptomatology [46], facilitating a quick understanding of the participant’s condition and the development of effective rapport.

#### Aim 2: Relationship Between Patient Engagement Levels and Symptom Controllability

Our study highlighted the importance of socioaffective engagement in improving symptom control. This type of engagement involves expressing positive emotions, valuing nurse input, using the nurse’s name, sharing personal experiences, showing vulnerability, and seeking ongoing interactions. These actions help build trust and foster a strong nurse-patient relationship, which is essential for effective online communication, learning, coordination, and symptom management.

The Nurse WRITE program supported this engagement through features such as nurse bios, photos, personalized web pages, and empathetic communication training. Future provider-guided DHIs could further enhance these efforts by accommodating participants’ communication preferences, such as voice-to-text conversion, and synchronous options, such as chat rooms, phone calls, text messages, and emails, to facilitate continuous interactions.

In the cognitive dimension, higher participant engagement significantly improved symptom control through effective coordination with nurses. Engagement included actions such as responding to questions, fulfilling requests, expressing agreement or disagreement, seeking information, and asking clarifying questions. This collaboration fostered knowledge sharing and the personalization of care plans, ultimately boosting patient confidence in managing symptoms post intervention.

Our study revealed that participants completing more care plans managed their symptoms better. This process, involving 6 out of 7 intervention elements, enhanced patients’ symptom understanding, corrected misconceptions, and built knowledge and confidence in effective symptom management. While such step-by-step processes may yield greater control and engagement [47], this method requires patience and effort, and failing to find a solution can lead to frustration and disengagement. Our qualitative analysis showed that the majority of low-engagement participants disengaged after the introduction phase but before completing a care plan. Previous studies have also suggested a connection between DHIs that allow participants to choose their interaction style and increased engagement [48-50]. Therefore, future provider-guided DHIs should aim for a harmonious blend of protocol adherence and flexibility, such as allowing early care plans, collaborative adjustments, and visual progress tracking on the homepage to enhance meaningful behavioral engagement and prevent frustration.

Although nurses worked to tailor communication to patients’ needs, strict protocol adherence posed challenges. Future DHIs should address these limitations by introducing greater flexibility, simplifying content, and utilizing diverse formats and technologies, such as visual aids, serious games, AI chatbots, or self-learning materials, to enhance comprehension and sustain engagement.

High completion rates of intervention questions and requests in participant posts indicated effective collaboration with nurses, with comprehensive responses addressing multiple questions. On the other hand, low completion rates led to recurring queries and extended communication, causing participant frustration and delaying symptom management. In Nurse WRITE, some nurses enhanced interactions by numbering questions and providing personalized feedback tailored to participants’ progress, goals, and challenges. Future DHIs could enhance participant attention and efficiency by incorporating formatting elements like color or italics to highlight key questions. Additionally, AI tools could prompt participants to address unanswered questions or requests before submitting their posts, fostering more efficient and continuous interactions.

These findings should be interpreted within the context of a structured, provider-guided intervention. In broader real-world settings, variations in provider availability, workflow constraints, and access to technology may influence engagement differently. Future research is needed to examine how these engagement patterns and influencing factors translate to less controlled clinical, community-based, and rural environments.

### Limitations

This exploratory study was limited by an analytic sample of 68 participants drawn from the larger Nurse WRITE intervention arm. The full eligible sample was not coded because multidimensional engagement analysis required intensive manual review and classification of asynchronous nurse-participant communication. Although the use of the first 68 eligible participants may limit generalizability and introduce potential selection bias, baseline comparisons showed no statistically significant differences between the analytic sample and the remaining eligible Nurse WRITE participants in demographic, psychosocial, or symptom-related variables. Despite this limitation, the study contributes to the limited evidence on socioaffective, cognitive, and behavioral engagement in provider-guided DHIs by applying a comprehensive engagement framework to evaluate patient factors, engagement processes, and intervention outcomes.

Second, multiple statistical tests were conducted across the 2 study aims, increasing the possibility of false positive findings. To address this issue, the Benjamini-Hochberg FDR correction was applied within each aim, and findings were interpreted in light of the adjusted results. Associations that did not remain statistically significant after FDR correction were considered exploratory and should be interpreted cautiously.

Third, the parent trial did not include direct measures of digital literacy. Therefore, although qualitative findings suggested that some participants experienced confusion with the intervention process, message length, website navigation, or computer-related tasks, we could not directly evaluate how literacy-related factors influenced engagement. Future studies should include digital literacy, health literacy, and communication preference measures to better identify participants who may need additional support.

Fourth, not all low engagers mentioned challenges or barriers before discontinuing communication on the message boards. However, as discussed in the Introduction, this situation was anticipated. To gain insights into their experiences and inform future provider-guided DHIs, we analyzed their intervention activities on the message boards before disengagement. Because these data were derived from intervention message boards rather than interviews designed specifically to explore disengagement, some reasons for disengagement may not have been fully captured.

Finally, generalizability may be limited because Nurse WRITE was a structured, nurse-guided intervention delivered to women with recurrent ovarian cancer in the context of a parent clinical trial. Engagement patterns may differ in other cancer populations, self-guided DHIs, less structured clinical programs, community-based settings, rural environments, or settings with different levels of provider availability and technology access.

### Conclusions

This study contributes to the limited evidence on socioaffective, cognitive, and behavioral engagement in provider-guided DHIs by applying a multidimensional engagement framework to evaluate patient factors, engagement processes, and intervention outcomes. Findings suggest that education was associated with engagement in specific cognitive, socioaffective, and behavioral intervention activities, while socioaffective and cognitive engagement were associated with improved perceived symptom control. These results underscore the need for provider-guided DHIs that balance protocol adherence with flexibility, simplify written communication, support early relationship-building, and accommodate participants’ communication preferences and symptom burden. Future research should validate these findings in larger and more diverse samples and examine how multidimensional engagement can be supported in real-world clinical, community-based, and rural settings.

## Supplementary material

10.2196/72784 Multimedia Appendix 1 Nurse WRITE (Written Representational Intervention To Ease Symptoms) intervention phases.

## Abbreviations

DHI: digital health intervention
FDR: false discovery rate
GRAMMS: Good Reporting of a Mixed Methods Study
HIPAA: Health Insurance Portability and Accountability Act
Nurse WRITE: Nurse Written Representational Intervention To Ease Symptoms
OR: odds ratio
STROBE: Strengthening the Reporting of Observational Studies in Epidemiology
