# Supplementary material for: Patient Engagement and Symptom Outcomes in a Provider-Guided Online Symptom Management Intervention: Mixed Methods Study
Source: J Med Internet Res. 2026 Jul 23;28:e72784. doi: 10.2196/72784 (PMC13395259; doi:10.2196/72784)
Supplement: Multimedia Appendix 1 [file jmir-v28-e72784-s001.docx]

## Multimedia appendices

### Appendix 1

| **Step** | **Intervention element** | **Description** |
| --- | --- | --- |
| 1 |  | The nurse initiates the message board by introducing herself and explaining how the intervention works on the message boards. The nurse then invited the patient to talk about herself and her experience with ovarian cancer and symptoms in general. |
| 2 | Representational assessment | The nurse proceeds to ask protocolized questions in the first few messages. This is for both the patient and the nurse to understand the patient’s experience with each symptom fully. As the patient thinks about the questions and writes responses, she may see some things more clearly or differently. A few examples of the protocolized questions include:   - What does your fatigue feel like, and how severe is it? - How has this symptom affected your life? Are you unable to do anything because of it? - How does your fatigue affect you emotionally? |
| 3 | Identifying and exploring Gaps, Errors, and Confusions | Throughout interactions with the participant, the nurse keeps attending to evidence of any confusion, concerns, or misconceptions about symptom management that the patient states or suggests during her message board posts. Any of these constitutes a barrier to effective symptom management for participants. |
| 4 | Creating conditions for conceptual change | Suppose any concerns/ misconceptions/gaps are identified. In that case, the nurse will (1) discuss the relationship between identified concerns/confusion/misconceptions and consequences of poor symptoms that the patient discussed during representational assessment and (2) provide information to address/counter the patient’s concerns, gaps, or misconceptions. |
| 5 | Introducing replacement information | The nurse will then guide the patient to read the relevant clinical practice guidelines and provide relevant information to choose and use strategies that fit well into their life and needs for managing symptoms. |
| 6 | Summary | The nurse will summarize information and how she believes it can benefit the patient. For example, it increases comfort and lessens interference with life. |
| 7 | Goal Setting and Planning | The nurse will encourage the patient to work together on developing symptom goals and specific strategies to reach those goals. |
| 8 | Goal and Strategy Review | After two weeks, the nurse will ask protocolized questions to work with the patient to evaluate strategies, reinforce success, and outline modifications as needed. The nurse will also discuss any barriers to implementing strategies and work with the patient to identify new or different strategies that could be integrated into her life. A few examples of the protocolized questions include:   - Were you able to use the above strategies? - If not, what things prevented you from doing so? - If yes, how well did the strategies (name/list strategies) help you reach your goal? |
| The back-and-forth discussion introduces the patient to a problem-solving approach to symptom management that could be applied to any symptom, not just the three they were working on. | | |
